# Myocardial Perfusion Imaging with Cardiovascular Magnetic Resonance in Nonischemic Cardiomyopathies: An In-Depth Review of Techniques and Clinical Applications

**DOI:** 10.3390/medicina61050875

**Published:** 2025-05-10

**Authors:** Ilir Sharka, Giorgia Panichella, Chrysanthos Grigoratos, Matilda Muca, Carmelo De Gori, Petra Keilberg, Giovanni Novani, Valerio Barra, Hana Hlavata, Matteo Bianchi, Denisa Simona Zai, Francesca Frijia, Alberto Clemente, Giancarlo Todiere, Andrea Barison

**Affiliations:** 1 Department of Cardiology, University Hospital “Mother Teresa”, 1005 Tirana, Albania; ilirsharka@gmail.com; 2 Department of Experimental and Clinical Medicine, University of Florence, 50121 Florence, Italy; 3 Cardiology and Cardiovascular Medicine, Fondazione Toscana Gabriele Monasterio, 54100 Pisa, Italy; cgrigoratos@ftgm.it (C.G.); todiere@ftgm.it (G.T.); abarison@ftgm.it (A.B.); 4 Department of Radiology, Fondazione G. Monasterio CNR-Regione Toscana, 56124 Pisa, Italy; mmuca@monasterio.it (M.M.); cdegori@ftgm.it (C.D.G.); petra.keilberg@ftgm.it (P.K.); novani@monasterio.it (G.N.); valeriobarra@monasterio.it (V.B.); hhlavata@monasterio.it (H.H.); mbianchi@monasterio.it (M.B.); dszai@monasterio.it (D.S.Z.); clemente@ftgm.it (A.C.); 5 Bioengineering Unit, Fondazione Toscana G. Monasterio, 56124 Pisa, Italy; ffrijia@ftgm.it

**Keywords:** cardiomyopathies, cardiovascular magnetic resonance, perfusion, ischemia, hypertrophic cardiomyopathy, fibrosis, microvascular dysfunction

## Abstract

*Background and Objectives*: Nonischemic cardiomyopathies comprise a wide spectrum of heart muscle disorders characterized by different morphological, functional, and tissue abnormalities. Cardiovascular magnetic resonance (CMR) represents the gold standard imaging modality for assessing cardiac morphology, systolic function, and tissue characterization, thereby aiding in early diagnosis, precise phenotyping, and tailored treatment. The aim of this review is to provide an up-to-date overview of CMR techniques for studying myocardial perfusion and their applications to nonischemic cardiomyopathy, not only to rule out an underlying ischemic aetiology but also to investigate the pathophysiological characteristics of microcirculatory dysfunction in these patients. *Materials and Methods*: We performed a structured review of the literature focusing on first-pass gadolinium perfusion sequences, stress protocols, and emerging pixel-wise perfusion mapping approaches. Studies were selected to illustrate the methods for image acquisition, post-processing, and quantification of myocardial blood flow (MBF) and myocardial perfusion reserve (MPR), as well as to highlight associations with clinical endpoints. *Results*: First-pass CMR perfusion imaging reliably detects diffuse and regional microvascular dysfunction across cardiomyopathies. Semi-quantitative parameters (e.g., upslope, MPRI) and quantitative MBF mapping (mL/g/min) have demonstrated that impaired perfusion correlates with disease severity, extent of fibrosis, and adverse outcomes, including heart failure hospitalization, arrhythmias, and mortality. Novel automated pixel-wise mapping enhances reproducibility and diagnostic accuracy, distinguishing coronary microvascular dysfunction from balanced three-vessel disease. Microvascular dysfunction—present in approximately 50–60% of dilated cardiomyopathy (DCM), 40–80% of hypertrophic cardiomyopathy (HCM), and >95% of cardiac amyloidosis (CA) patients—has emerged as a key driver of adverse outcomes. Perfusion defects appear early, often preceding overt hypertrophy or fibrosis, and provide incremental prognostic value beyond conventional CMR metrics. *Conclusions*: CMR represents a powerful tool for detecting myocardial perfusion abnormalities in nonischemic cardiomyopathies, improving phenotyping, risk stratification, and personalized management. Further standardization of quantitative perfusion techniques will facilitate broader clinical adoption.

## 1. Introduction

Cardiomyopathies are a group of myocardial disorders, often affecting young individuals, characterized by the presence of structural and functional abnormalities of the heart muscle that are not explained by coronary artery disease (CAD), hypertension, valvular disease, or congenital heart disease [1]. The non-invasive characterization of cardiomyopathies has received a significant boost from recent advances in cardiovascular magnetic resonance (CMR) imaging, which currently represents the gold standard for the non-invasive assessment of cardiac morphology, function, and myocardial tissue abnormalities. In fact, CMR allows not only the quantification of biventricular volumes, mass, wall thickness, and systolic and diastolic function, as well as intra- and extracardiac flows, but also the detection of myocardial edema, fibrosis, and the accumulation of other intra/extracellular substances (such as fat, iron, and amyloid). This provides unique information for the etiological, diagnostic, and prognostic definition of the disease [1].

Myocardial perfusion is usually overlooked in nonischemic cardiomyopathies; however, several studies have demonstrated that microvascular dysfunction plays a significant role in the pathophysiology of these conditions. Impaired myocardial blood flow has been observed in various forms of nonischemic cardiomyopathy, even in the absence of epicardial CAD. Reduced perfusion reserve, often caused by microvascular remodeling, capillary rarefaction, or endothelial dysfunction, has been linked to disease progression, adverse remodeling, arrhythmias, and an increased risk of heart failure (HF). CMR myocardial perfusion imaging (MPI), particularly when combined with quantitative perfusion analysis, allows for the non-invasive assessment of microvascular integrity and has the potential to enhance risk stratification and guide therapeutic strategies in these patients.

**Figure 1:**
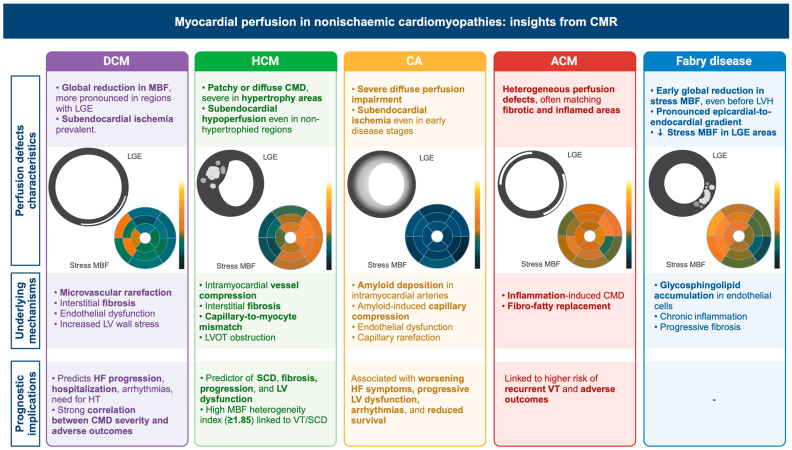
Myocardial perfusion in nonischemic cardiomyopathies: insights from cardiovascular magnetic resonance (CMR). The figure summarizes the perfusion characteristics, underlying mechanisms, and prognostic implications of myocardial perfusion abnormalities in various forms of nonischemic cardiomyopathy, as assessed using CMR. Each cardiomyopathy is represented with an illustration of late gadolinium enhancement (LGE) and stress myocardial blood flow (MBF) patterns. Perfusion abnormalities are linked to distinct structural and microvascular alterations that contribute to disease progression, arrhythmogenesis, and clinical outcomes. ACM, arrhythmogenic cardiomyopathy; CA, cardiac amyloidosis; CMD, coronary microvascular dysfunction; CMR, cardiovascular magnetic resonance; DCM, dilated cardiomyopathy; HCM, hypertrophic cardiomyopathy; HF, heart failure; HT, heart transplantation; LV, left ventricle; LGE, late gadolinium enhancement; LVH, left ventricular hypertrophy; LVOT, left ventricular outflow tract; MBF, myocardial blood flow; SCD, sudden cardiac death; VT, ventricular tachycardia.

The aim of this review is to provide a comprehensive overview of the role of CMR in assessing myocardial perfusion in nonischemic cardiomyopathies, exploring its potential to improve the understanding of disease mechanisms, optimize clinical decision-making, and refine risk stratification in affected patients (Figure 1).

## 2. Stress Cardiac Magnetic Resonance

**Figure 2:**
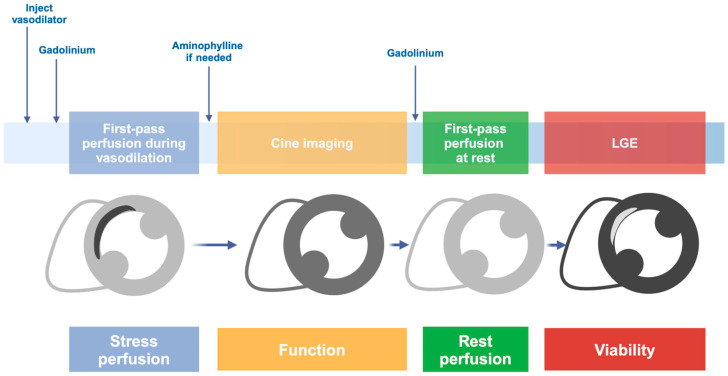
Stress CMR protocol with vasodilator. The process begins with stress perfusion (1–6 min, *blue section*), during which a vasodilator is administered to induce hyperemia, simulating stress conditions, and gadolinium contrast is injected to evaluate myocardial blood flow and detect stress-induced ischemia. This is followed by aminophylline injection, if needed, to counteract vasodilator effects and by cine imaging (15 min, *yellow section*), during which cardiac function and wall motion abnormalities are assessed. Next, rest perfusion (2 min, *green section*) is performed with a second gadolinium injection, serving as a baseline to identify perfusion defects at rest and distinguish ischemia from fixed defects. The final step, late gadolinium enhancement (LGE) (5–10 min, *red section*), evaluates myocardial viability by detecting areas of necrosis or fibrosis. The bottom row visually represents the progression of myocardial perfusion assessment, from stress-induced ischemia to function evaluation, resting ischemia detection, and viability assessment.

**Figure 3:**
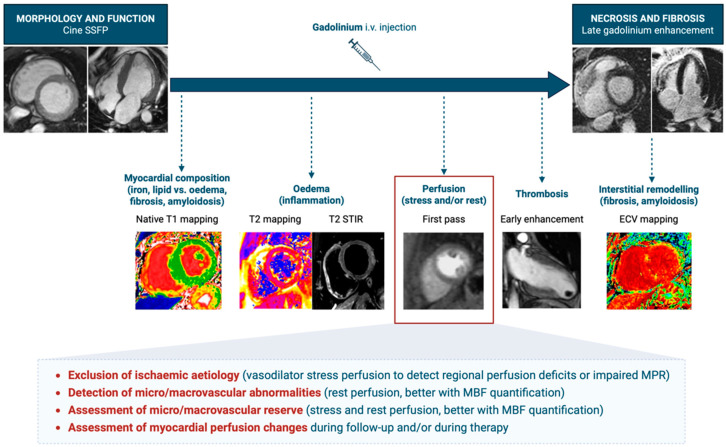
Cardiac magnetic resonance scan workflow in nonischemic cardiomyopathies. Please note that cine SSFP images can also be acquired between stress and rest perfusions (as represented in Figure 2) or between early and late enhancement imaging, depending on the scanning protocol. ECV, extracellular volume; MBF, myocardial blood flow; MPR, myocardial perfusion reserve; SSFP, steady-state free precession; STIR, short-tau inversion recovery.

Stress CMR is a recommended imaging modality for diagnosing and assessing prognosis in patients with chronic stable angina. Compared to more commonly used techniques, like single-photon emission computed tomography (SPECT), stress CMR offers several technical advantages, including superior spatial resolution, a wider field of view, and enhanced tissue differentiation [2]. These features help eliminate attenuation artifacts and prevent misleading perfusion signals from non-cardiac structures, which can otherwise obscure or mimic perfusion defects. Additionally, it is non-invasive and does not involve ionizing radiation, making it a safer and more patient-friendly option. Like other imaging methods, ischemia is evaluated through myocardial perfusion and wall motion analysis under pharmacological vasodilation (using adenosine, dipyridamole, or regadenoson) or inotropic/chronotropic stimulation with dobutamine. Stress acquisitions are then complemented by rest acquisitions: rest perfusion imaging serves as a reference to identify fixed perfusion defects (either due to a previous scar or an artifact); rest cine imaging is used as a baseline for assessing wall motion abnormalities; and late gadolinium enhancement (LGE) is employed to evaluate myocardial viability (Figure 2). In patients with nonischemic cardiomyopathies, stress CMR is one of the non-invasive imaging techniques used to rule out an ischemic etiology [1], as well as to better characterize perfusion reserve. In addition to the established role of CMR in providing detailed morphological, functional, and tissue characterization of the heart with dedicated sequences, myocardial perfusion imaging (using gadolinium first-pass sequences acquired at rest and/or during stress) provides unique information on macro- and microvascular function (Figure 3). Several CMR sequences have been developed to assess myocardial perfusion, based on different pulse and readout schemes.

Early perfusion techniques focused on the first pass of gadolinium-based contrast agents, offering insight into myocardial blood flow (MBF). Images are captured during the rapid transit of contrast through the myocardium following intravenous administration. Initial studies used fast gradient-echo (FGE) sequences, providing some of the first contrast-enhanced images of myocardial perfusion [3,4]. However, challenges such as low spatial and temporal resolution, signal saturation, and motion sensitivity are significant limitations [5]. The introduction of steady-state free precession (SSFP) sequences improved image quality by providing a higher signal-to-noise ratio and better contrast between the myocardium and blood pool. However, SSFP acquisitions were more prone to dark-rim artifacts compared to FGE sequences [6].

On the backbone of cine steady-state free precession (SSFP) imaging for morphological and functional assessment and late gadolinium enhancement for necrosis and fibrosis detection, several other sequences may be added according to the clinical indication. Native T1 mapping is a quantitative parametric sequence used to estimate global myocardial tissue composition, where iron or fat accumulation shortens native T1, while edema, fibrosis, and amyloid increase native T1. T2 mapping and short tau inversion recovery (T2-STIR) sequences are used to detect myocardial intracellular and extracellular edema, which is usually correlated with the presence of myocardial inflammation. First-pass sequences are acquired soon after contrast injection, either at rest or during stress, to assess myocardial perfusion in different pathophysiological states. Early enhancement sequences are acquired 1–2 min after contrast injection, with an inversion time set to null intracardiac thrombosis. Extracellular volume (ECV) mapping is calculated from late post-contrast T1 mapping, after correction for native T1 and blood hematocrit, to quantify myocardial interstitial remodeling, which is tightly correlated with the extracellular burden of collagen, amyloid, or other substances. Different modules can be chosen according to the clinical questions, the patient’s clinical history, and other technical factors (patient cooperation, total scan duration, presence of arrhythmias/electronic devices, contraindications to gadolinium, contraindications to stress agents).

The adoption of saturation recovery pulse sequences further enhanced temporal resolution and image consistency by allowing for magnetization recovery between acquisitions [7]. Initially, qualitative analysis of first-pass perfusion imaging was the standard approach, in which clinicians visually assessed dynamic perfusion images for defects. However, this method is highly dependent on observer expertise, leading to significant variability in diagnostic accuracy and inter-reader agreement [8]. The subjective nature of visual assessments makes it difficult to detect subtle perfusion abnormalities and accurately quantify ischemic burden, potentially impacting clinical decision-making [9].

To overcome these limitations, the field has increasingly shifted towards semi-quantitative CMR perfusion imaging, which provides objective measurements of MBF [10]. The semi-quantitative assessment of myocardial perfusion has evolved from research applications to clinical practice. Researchers developed signal intensity curve analysis to derive semi-quantitative parameters such as the maximal upslope, time-to-peak, and myocardial perfusion reserve index (MPRI), which is the ratio of stress/rest upslope. These metrics have proven useful for detecting CAD and coronary microvascular dysfunction (CMD) [11,12]. However, variability in acquisition protocols and analysis techniques has led to inconsistent diagnostic performance, highlighting the need for standardization [11,12].

Fully quantitative MBF measurements (expressed in mL/g/min) are derived using time-intensity curve deconvolution with an arterial input function (AIF) as a reference. Two primary strategies are used for AIF measurement: (1) the “dual-bolus” method, which involves a separate low-dose injection (1/10th or 1/20th of the full dose) before the main contrast injection; and (2) the “dual-sequence” method, which dedicates one imaging slice for AIF measurement by applying a short saturation recovery time. Both approaches mitigate signal saturation effects that arise from high contrast agent concentrations during bolus transit [13]. Additionally, proton density-weighted imaging techniques have been employed to correct for surface coil intensity bias [14]. Quantitative CMR perfusion imaging now offers precise MBF measurements, improving the detection of ischemia and CMD. Recent advancements include semi-automated and fully automated pixel-wise MBF mapping, which enhance clinical integration and workflow efficiency [15]. In patients with CMD, a diffuse subendocardial perfusion defect is typically visible, often indistinguishable from balanced three-vessel coronary artery disease. Based on specific MBF thresholds, quantitative myocardial perfusion mapping has been shown to distinguish CMD from three-vessel coronary artery disease with a high level of accuracy. In a study by Kotecha et al. [10], patients with CMD presented either a global stress MBF of <2.25 mL/g/min without any regional perfusion defect or a regional CMD perfusion defect with preserved regional stress MBF (>1.94 mL/g/min).

In summary, the evolution from qualitative to quantitative CMR perfusion imaging represents a significant advancement in cardiac diagnostics, addressing the challenges associated with early qualitative methods and paving the way for more accurate and effective patient care. Currently, while CMR excels at high-resolution fibrosis mapping (LGE, T1/ECV) and pixel-wise perfusion, PET still represents the gold-standard imaging modality for absolute perfusion quantification. A comparison of different noninvasive imaging modalities to assess myocardial perfusion is provided in Table 1.

### 2.1. Stress CMR with Vasodilators

Vasodilator drugs share the common ability to bind to adenosine receptors, but they differ in receptor selectivity, half-life, and duration of action [16].

Adenosine is administered intravenously (i.v.) at a dose of 140–210 μg/kg/min for 4–6 min. It has a half-life of less than 10 s and a very short duration of action (a few seconds). While selective activation of A2A receptors induces coronary vasodilation, additional effects on A1 and A2B receptors can lead to side effects such as atrioventricular block (AVB) and bronchospasm, respectively [2].

Dipyridamole is administered at a dose of 0.56–0.84 mg/kg i.v. over 6 min and has a longer half-life (approximately 11 h) and duration of action (about 30 min) compared to adenosine. However, it shares similar receptor selectivity issues and potential side effects, including AVB, breathlessness, bronchospasm, hypotension, headache, and flushing. As a result, adenosine and dipyridamole should be avoided in patients with severe sinus bradycardia (<40 beats per minute), advanced AVB, asthma, or severe reactive pulmonary disease requiring chronic inhaler therapy [2].

Regadenoson is administered as a fixed-dose bolus of 400 μg over 10 s, followed by a 10 s saline flush. It has a relatively short half-life (30 min) and a duration of action of approximately 2.3 min. Due to its selective activation of A2A receptors, it provides effective coronary vasodilation while minimizing side effects and improving patient tolerability. Notably, regadenoson can be safely used in patients with asthma or reactive airway disease and has a lower incidence of AVB [2].

During intense vasodilation, myocardial regions supplied by stenotic coronary arteries exhibit relative hypoperfusion, appearing as hypointensity during first-pass perfusion. Wall motion abnormalities become apparent only when coronary stenosis is severe enough to cause ischemia, leading to an absolute reduction in blood flow due to the “coronary steal” phenomenon [17].

To maintain the diagnostic sensitivity of the test, patients should abstain from consuming coffee, tea, or other caffeine-containing beverages (including some “decaffeinated” drinks that may still contain caffeine) for at least 24 h before undergoing CMR, as caffeine can block the effects of vasodilators.

Beyond qualitative analysis, myocardial perfusion reserve (MPR) can be assessed using a semiquantitative approach that calculates the ratio between the myocardial signal intensity maximal upslope at peak stress and at rest, both normalized to the blood pool signal intensity. Additionally, quantitative myocardial perfusion estimation is now feasible with recent imaging sequences based on either the dual gadolinium bolus technique or the dual-sequence technique, which incorporates both myocardial and AIF measurements [18].

### 2.2. Stress CMR with Dobutamine

Functional stress CMR is generally based on the administration of dobutamine, a synthetic catecholamine with a predominant affinity for beta-1 adrenergic receptors [16]. The demonstration of myocardial ischemia using the dobutamine stress test relies on an increase in myocardial oxygen demand, which is caused by positive inotropic and chronotropic effects. Low-dose dobutamine stress is used to assess myocardial inotropic reserve and viability (based on the assumption that stunned regions are recruited), typically in patients with ischemic or nonischemic left ventricular dysfunction. High-dose dobutamine, on the other hand, is used to simulate maximal physiological effort and to test for the presence of myocardial ischemia, which can be detected as either wall motion or perfusion regional abnormalities.

Dobutamine is administered at increasing doses, up to a maximum of 40 μg/kg/min i.v. (±atropine), and has a half-life of 1–2 min [19]. Cine images are acquired at each step, and perfusion imaging can also be obtained at peak stress for comparison with rest perfusion. Potential side effects include palpitations, shortness of breath, headache, and supraventricular and ventricular arrhythmias. Dobutamine stress CMR should not be performed in cases of severe aortic stenosis, obstructive hypertrophic cardiomyopathy (HCM), or complex arrhythmias. Beta-blockers should be discontinued 48 h prior to dobutamine administration due to their competing pharmacological action [19].

### 2.3. Contraindications and Safety Considerations

Common contraindications for both vasodilator and inotropic stressors include unstable angina, acute myocardial infarction, severe systemic arterial hypertension (≥220/120 mmHg), and other serious acute clinical conditions. Although most adverse effects are self-limiting, i.v. aminophylline can serve as an antidote for vasodilator stress testing, whereas i.v. beta-blockers can be used to reverse the effects of dobutamine.

Due to the potential for clinically relevant side effects or the induction of severe myocardial ischemia during a stress test, all magnetic resonance staff should continuously monitor heart rhythm, blood pressure, and symptoms, and should be regularly trained in the recognition and management of acute cardiovascular emergencies [16] in accordance with international guidelines on basic and advanced life support [20].

## 3. Dilated Cardiomyopathy

Non-ischemic dilated cardiomyopathy (DCM) is characterized by a dilated and poorly contractile left and/or right ventricle, resulting from a complex interplay between genetic predisposition and environmental factors [21]. CMR is considered the gold standard imaging technique for quantifying chamber volumes, myocardial mass, and ejection fraction. In addition to its structural assessment capabilities, CMR enables the detection of myocardial fibrosis, which is recognized as having significant prognostic value in DCM [22,23].

### 3.1. Non-CMR Studies on Myocardial Perfusion in DCM

Several non-invasive cardiac imaging modalities have traditionally been used to assess perfusion abnormalities in DCM, including echocardiography, single-photon emission computed tomography (SPECT), and positron emission tomography (PET).

Echocardiography has provided key insights into the pathophysiology and prognosis of myocardial perfusion in nonischemic DCM. These studies primarily focus on the evaluation of coronary flow reserve (CFR), which is assessed using Doppler quantification of the peak stress-to-rest velocity ratio in the left anterior descending artery (LAD) [24,25,26]. Another important parameter is MPR, which reflects microcirculatory function and is assessed using myocardial contrast echocardiography [27]. Studies analyzing both CFR and MPR simultaneously have shown that impaired myocardial perfusion is a common feature in DCM [28]. Reduced perfusion is associated with higher myocardial wall stress, increased pulmonary capillary wedge pressure, elevated left ventricular (LV) end-diastolic pressure, and a lower LV ejection fraction (LVEF) [24]. Additionally, impairment in CFR and MPR has been linked to the absence of contractile reserve [25,26]. Beyond its pathophysiological implications, reduced myocardial microcirculation perfusion is predictive of disease severity and clinical outcomes. Decreased perfusion correlates with impaired myocardial strain indices [29] and has been shown to predict the response to optimal medical therapy [30]. Furthermore, CFR and MPR have been identified as independent predictors of cardiac death and the need for heart transplantation in nonischemic DCM, providing incremental prognostic value beyond traditional clinical and echocardiographic factors [28].

SPECT imaging has shown that up to 97% of nonischemic DCM patients exhibit myocardial perfusion defects, most commonly in the septal wall [31]. About half show reversible defects, while in one-third, non-reversible defects correspond to myocardial fibrosis, as confirmed by LGE [31]. Studies on perfusion defect patterns in nonischemic DCM identify two groups: those with large focal defects and those with non-focal impairments, which appear as minimal or multiple small defects. Patients with large focal defects tend to have higher natriuretic peptide and plasma norepinephrine levels, longer QRS durations, more frequent ventricular arrhythmias, and a higher HF functional class, contributing to a worse prognosis and increased mortality compared to those with non-focal defects [32]. Mild fixed defects, especially in the inferior wall, are occasionally observed in nonischemic DCM, likely due to attenuation artifacts from severe LV dilation and supine imaging [33]. In nonischemic DCM with conduction abnormalities like left bundle branch block, SPECT imaging may show heterogeneous 99mTc-MIBI uptake due to regional wall thickening differences [34,35]. However, this does not always reflect true myocardial blood flow; rather, it represents a partial-volume effect that can mimic septal hypoperfusion, leading to misinterpretation. Additionally, regions with impaired wall thickening exhibit a higher technetium-99m methoxyisobutylisonitrile (99mTc-MIBI) washout rate, indicating metabolic and mitochondrial dysfunction severity. Since 99mTc-MIBI uptake depends on mitochondrial and plasma membrane potentials, its distribution provides insights into both coronary perfusion and oxidative dysfunction [34,35].

Unlike SPECT, which estimates LV function and perfusion, PET imaging offers superior resolution and enables absolute quantification of MBF and CFR, making it particularly useful for detecting subtle CMD and differentiating ischemic from nonischemic cardiomyopathies [36,37]. PET plays a key role in identifying CMD, which is common in nonischemic DCM, correlates with LV dysfunction severity, and can be detected before symptomatic HF onset, carrying a poor prognosis [38,39]. The ability to identify CMD using PET opens new possibilities not only for early diagnosis but also for disease monitoring [40]. PET-MPI studies suggest that impaired myocardial perfusion is linked to a regional mismatch between blood flow and oxygen consumption, increased LV wall stress, and potential ischemia [41]. Additionally, reduced peak hyperemic MBF and CFR strongly predict HF progression, mortality, and major cardiac events, including HF hospitalization and sudden cardiac death (SCD) [42,43,44]. Impaired myocardial perfusion reserve and increased coronary resistance have also been noted in non-ischemic DCM patients with atrial fibrillation, further highlighting the clinical importance of perfusion assessment [45].

### 3.2. CMR Studies on Myocardial Perfusion in DCM

Recent studies on myocardial perfusion in non-ischemic DCM using CMR have provided significant insights into the pathophysiology and prognostic implications of this condition (Table 2). Although reduced transmural MBF at baseline and during hyperemia has been demonstrated by PET in patients with non-ischemic DCM, PET has limitations in differentiating subendocardial from subepicardial flow. In contrast, first-pass contrast-enhanced CMR allows for the direct visualization of subendocardial and subepicardial perfusion abnormalities [18] and has been validated against microsphere-assessed blood flow measurements [14].

DCM is generally characterized by a global reduction in MBF, which is more pronounced in the subendocardium and in myocardial areas with LGE. The impairment of subendocardial perfusion reserve and oxidative metabolism observed in non-ischemic DCM is consistent with the concept of subendocardial energy starvation in HF [46]. Both semi-quantitative and fully quantitative CMR perfusion studies have confirmed the presence of impaired stress MBF and MPR, indicating the presence of CMD [47]. Furthermore, these studies have demonstrated that CMD is associated with major adverse cardiovascular events, including HF hospitalization, ventricular arrhythmias, and cardiovascular death, independent of LVEF and indexed ventricular volumes [48]. By utilizing oxygenation-sensitive CMR and magnetic resonance spectroscopy (MRS), additional insights have been gained into the pathophysiological mechanisms of DCM. These studies have revealed a dissociation between CMD and myocardial oxygenation, indicating that cardiac energetics remain unaffected by oxygen supplementation. This suggests the absence of clinically relevant myocardial hypoxia at rest in non-ischemic DCM [49].

The relationship between stress and rest MBF in DCM has also been explored, revealing that myocardial segments with LGE exhibit lower perfusion compared to LGE-negative regions. This suggests that myocardial fibrosis is associated with impaired perfusion and that the severity of CMD correlates with the degree of LV impairment. If CMD plays a causative role in DCM pathogenesis, it is more likely to involve stress-induced repetitive stunning rather than chronic myocardial hypoperfusion [50].

The diagnostic accuracy of high-resolution quantitative perfusion CMR has been validated against invasive CFR measurements. Studies have established optimal diagnostic thresholds for detecting CMD, including a global MPR cutoff of 2.19, with subendocardial MPR thresholds reaching 2.41 [51]. However, for widespread clinical implementation, the standardization of CMR perfusion protocols remains essential due to significant inter-study heterogeneity, variability in acquisition techniques, and differences in perfusion parameter assessment methods [12].

The multifaceted parametric approach of CMR has provided valuable advancements in the diagnosis, risk stratification, and management of DCM. T1 mapping and extracellular volume fraction (ECV) measurements have emerged as key techniques for assessing interstitial myocardial fibrosis. Increased native T1 and ECV values have been associated with HF progression and arrhythmia-related events, serving as independent predictors of adverse outcomes and offering a more comprehensive risk stratification tool beyond conventional parameters such as LVEF [52]. In addition, CMR feature tracking has been utilized to evaluate LV strain abnormalities in DCM. Studies have demonstrated significant reductions in global peak radial, circumferential, and longitudinal strain, with global longitudinal strain showing the strongest predictive value for worsening HF, hospitalizations, and HF-related death [53].

The integration of advanced CMR techniques, including LGE, T1 mapping, and the MPR index, provides a comprehensive prognostic framework for DCM evaluation. These methods allow for detailed myocardial tissue characterization, which is crucial for guiding therapeutic decisions and improving patient outcomes [54,55].

**Table 2:** Studies on myocardial perfusion in non-ischemic cardiomyopathies.

Author, Year [Ref]	Patients	Stressor	Quantitative (Yes/No)	Main Finding	Prevalence of Perfusion Defects	Distribution of Perfusion Defects
**Dilated cardiomyopathy**						
Bell SP, 2013 [46]	13 NIDCM pts/15 controls in CMR and PET study	adenosine	No	- Impaired subendocardial perfusion reserve (MPRI) in NIDCM - Impaired oxidative metabolism	All	Circumferential subendocardial perfusion defects (hypoperfusion)
Dass S, 2015 [49]	14 NIDCM pts/12 controls in CMR, OS-(BOLD) CMR, spectroscopy MR	adenosine	No	- Reduced MPRI in NIDCM - No change in oxygenation between groups - No change in cardiac energetics by oxygen supplementation - Dissociation between MVD and oxygenation	-	Circumferential perfusion defects
Gulati A, 2019 [50]	65 NIDCM pts/45 controls	adenosine	yes	- Lower stress MBF and impaired MPR in NIDCM	-	Circumferential perfusion defects
Slivnick JA, 2022 [47]	41 NIDCM pts/58 controls	adenosine	No	- Impaired MPRI in NIDCM - MPRI superior to visual analysis for MVD detection	56% (by MPRI) 27% (visually)	Subendocardial stress perfusion defects
Javed W, 2023 [48]	160 NIDCM pts	adenosine	Yes	Reduced MPR predicted MACE	42/160 pts (26%) with MPR < 2.06	Subendocardial perfusion defects
**Hypertrophic cardiomyopathy**						
Petersen SE, 2007 [56]	35 HCM pts/14 controls	adenosine	yes	- Reduced stress MBF in proportion to magnitude of hypertrophy - MVD and subsequent ischemia as components of risk attributable to HCM	-	Predominatly subendocardial perfusion defects
Ismail TF, 2014 [57]	35 HCM pts	adenosine	yes	Pixel-wise quantitative CMR perfusion differentiates HCM pts with non-severe from severe localized MVD (potential myocardial ischemia)	- 31.4% (11 pts) with severe MVD - 68.6% (24 pts) with non-severe MVD	Predominantly subendocardial perfusion defects
Xu H, 2014 [58]	42 HCM pts/14 controls	-	No	- Regional MVD more pronounced in obstructive vs. non-obstructive HCM (also in hypetrophic vs. non-hypertrophic segments).	3 pts (12%) in obstructive HCM group	Predominantly subendocardial perfusion defects
Chiribiri A, 2015 [59]	80 HCM pts	-	No	- Abnormal rest perfusion in a significant proportion of HCM pts - Rest perfusion abnormalities identify high risk pts (prevalence of NSVT > twice as high in fibrosis+/Perfusion+ group)	24 pts (30%) with rest perfusion defets in visual analysis	Predominantly subendocardial perfusion defects
Villa ADM, 2016 [60]	30 HCM pts	adenosine	yes	- LGE—important confounder in assessment of ischemia burden - High-resolution analysis of fibrosis and perfusion enables independent evaluation of microvascular ischemia and fibrosis	Visual analysis: - stress-induced perfusion abnormalities in 60% of pts	- Subendocardial perfusion defects (47% of pts) - Mixed perfusion defects (13% of pts) - Scar-related perfusion defects (3%)
Tezuka D, 2018 [61]	81 pts (37 HCM, 24 LVH, 20 normal controls) in stress CMR	adenosine	No	- Stepwise MPR decrease between LVH and HCM - Lower MPR in HCM with LGE than HCM without LGE	-	Predominantly subendocardial perfusion defects
Kim EK, 2020 [62]	115 HCM pts	adenosine	No	Stress perfusion defects associated with NSVT, higher LV mass index, apical aneurysms	42% of pts (visual analysis)	- Multiple patchy patterns (50%) - Concentric subendocardial pattern (41.7%) - Single blot-like defect (8.3%)
Camaioni C, 2020 [63]	101 HCM pts/30 controls	adenosine	yes	- MVD is common in HCM associated with hypertrophy and LGE - Impaired stress perfusion is an early disease marker	79% of pts with perfusion defects	- Predominantly subendocardial - Reduced MBF and MPR with increasing wall thickness and LGE, but also in apparently “normal” myocardial segments
Raman B, 2021 [64]	103 HCM/32 controls in stress CMR/OS-(BOLD) CMR	adenosine	yes	- 3 HCM pts groups (Sarc-HCM, Sarc + HCM, Sarc + VUS) have impaired stress perfusion and blunted oxygenation vs. controls	-	Predominantly subendocardial perfusion defects
Hughes RK, 2021 [65]	50 Genotype + LVH- pts/28 controls	adenosine	yes	Impaired myocardial perfusion in HCM mutation carriers even in the absence of significant LVH or scarring	9 pts (20%) of carriers with visual perfusion defects in perfusion mapping	Predominantly subendocardial perfusion defects
Das A, 2022 [66]	20 HCM pts in stress CMR/ 10 cDTI controls	adenosine	yes	Lower MPR and cardiomyocyte disarray in HCM	-	Mainly subendocardial perfusion defects
Garcia Brás P, 2023 [67]	75 HCM pts CMR and strain echo study	regadenoson	No	Impaired myocardial work significantly correlated with MVD (with higher predictive power than GLS); independently of LGE, LV obstruction and hypertrophy	90.7% of pts with perfusion defects	- Predominantly subendocardial perfusion defects - Perfusion defects: 22.5% ± 16.9% of LV mass (mean ± SD) - Segments with perfusion defects: 5.7 ± 3.9 (mean ± SD)
**Cardiac amyloidosis**						
Li R, 2016 [68]	32 AL amyloidosis pts/25 healthy controls	-	No	- AL-CA patients show reduced first-pass perfusion - (lower upslope and MaxSI, increased TTM)	-	- Subendocardial/diffuse perfusion impairment - Regional basal–apical gradient of reduced perfusion
Kotecha T, 2019 [69]	86 amyloidosis pts/20 healthy volunteers	adenosine	yes	- Myocardial ischemia common in CA - Reduced stress MBF and MPR early disease markers	-	Subendocardial/diffuse perfusion impairment
Ioannou A, 2022 [70]	92 amyloidosis pts	adenosine	yes	Reduced MPR comparable to 3 vessel disease pts	-	Subendocardial/diffuse perfusion impairment
Chacko L, 2024 [71]	93 amyloidosis pts (42 and 51 ATTR)/97 controls (74 3VD)	adenosine	yes	CMR stress perfusion mapping and histology demonstrate severe inducible myocardial ischemia in CA	-	Subendocardial/diffuse perfusion impairment
Katznelson E, 2024 [72]	92 AL amyloidosis pts in rest CMR	-	yes	MBF and MWE decrease as cardiac amyloid burden (ECV) increase	-	Subendocardial/diffuse perfusion impairment
Tang L, 2025 [73]	126 AL amyloidosis pts	adenosine	yes	ECV and MPR show high prognostic value (higher ECV and lower MPR—worse prognosis)	-	Subendocardial/diffuse perfusion impairment
**Arrhythmogenic cardiomyopathy**						
Tung R, 2015 [74]	103 pts in a PET/CMR study	-	no	Near 50% of unexplained cardiomyopathy and VA demonstrate inflammation, fibrotic remodeling and microvascular dysfunction	Perfusion defects present in - 52% of AIC+ - 48% of AIC	Heterogeneous matching fibrotic and inflamed areas
**Fabry disease**						
Knott KD, 2019 [75]	44 Fabry pts/27 healthy controls	adenosine	yes	- FD patients have reduced perfusion (particularly subendocardial with LVH) - Impaired perfusion—early disease marker	-	Predominantly subendocardial perfusion impairment

3VD, three-vessel disease; AIC+, arrhythmogenic inflammatory cardiomyopathy plus systemic inflammation; BOLD, blood oxygen level-dependent; CA, cardiac amyloidosis; ECV, extracellular volume; FD, Fabry disease; GLS, global longitudinal strain; HCM, hypertrophic cardiomyopathy; LVH, left ventricular hypertrophy; MACE, major adverse cardiovascular events; MaxSI, maximal signal intensity; MPRI, myocardial perfusion index; MVD, microvascular dysfunction; MWE, maximal work efficiency; NIDCM, non-ischemic dilated cardiomyopathy; NSVT, non sustained ventricular tachycardia; OS, oxygenation sensitive; Sarc, sarcomere gene mutation; TTM, time to maximum signal intensity; VA, ventricular arrhythmia; VUS, variant of unknown significance.

## 4. Hypertrophic Cardiomyopathy

Hypertrophic cardiomyopathy (HCM) is a genetic disorder characterized by inappropriate myocardial hypertrophy, myocardial fibrosis, and diffuse disarray, exhibiting diverse phenotypic expressions, clinical courses, and prognoses [76]. CMR is the gold standard imaging modality for assessing ventricular mass, volume, function, wall thickness, and tissue characterization, all without exposure to ionizing radiation [1,77].

CMR is particularly useful for detecting unusual patterns of myocardial hypertrophy, including LV lateral and apical distribution, right ventricular (RV) involvement, and papillary muscle hypertrophy. Moreover, CMR allows for the differential diagnosis between sarcomeric HCM and its phenocopies [78] and enables the assessment of myocardial fibrosis [79]. A LGE threshold of 10–15% of LV mass has been proposed as a cutoff for identifying patients at high risk of SCD who may benefit from primary prevention strategies, even in the absence of other major risk factors [77].

Several studies have highlighted the presence of impaired MBF and its underlying mechanisms in HCM. The interplay between reduced capillary density, caused by hypertrophy, and extravascular compressive forces contributes to CMD in these patients [80]. During systole, the compression of intramyocardial blood vessels results in an abnormally large backward compression wave, while severe LV outflow tract obstruction generates an additional deceleration wave, both of which lead to reduced coronary blood flow. Thus, perfusion abnormalities in HCM are not merely a consequence of supply–demand mismatch or remodeling of intramyocardial vessels but instead represent a dynamic interaction with the mechanics of myocardial ischemia [81].

### 4.1. Non-CMR Studies on Myocardial Perfusion in HCM

Vasodilator stress myocardial contrast echocardiography (MCE) studies in HCM patients with extensive fibrosis have revealed different spatial patterns of perfusion defects between hypertrophied and non-hypertrophied myocardial segments. The majority of septal perfusion defects appear patchy or subendocardial, whereas non-hypertrophied segments demonstrate transmural and diffuse perfusion defects, reflecting distinct structural and functional abnormalities [82]. MCE combined with PET and cardiac catheterization has shown that HCM patients exhibit lower resting and hyperemic MBF compared to controls, with LV end-diastolic pressure and LV mass index emerging as independent predictors of reduced myocardial perfusion [83]. Doppler echocardiography has further confirmed reduced CFR in stress vasodilator studies, which is considered an independent predictor of cardiovascular events in HCM [84].

Thallium-201 SPECT during dipyridamole stress testing has identified myocardial perfusion defects in HCM patients, with fixed perfusion defects associated with syncope. However, these defects do not correlate directly with disease-related mortality [85]. Reversible perfusion defects have been linked to increased left atrial dimensions and greater ventricular wall thickness, suggesting their potential role in assessing disease severity [85]. SPECT studies have demonstrated that myocardial perfusion defects serve as predictors of adverse clinical events, identifying patients at increased risk of cardiovascular death in both adult [86] and pediatric [87] HCM populations. The absence of perfusion defects in the anterior junctional area has been proposed as a marker of low-risk status in HCM patients [88].

PET-MPI has been considered one of the most effective techniques for assessing CMD in HCM [89]. Studies have further elucidated how CMD predisposes individuals to myocardial ischemia, cardiac dysfunction, and fibrosis. The severity of CMD and the associated mechano-energetic uncoupling play a central role in HCM pathogenesis and are linked to an increased risk of adverse clinical outcomes and mortality [90]. Regional heterogeneity of resting perfusion in HCM correlates with the extent of LGE in CMR but not with regional systolic function [91]. High heterogeneity of stress MBF, characterized by an MBF heterogeneity index of ≥1.85, has been identified as a PET biomarker for ventricular arrhythmias in symptomatic HCM patients [92]. Moreover, impaired stress MBF and an abnormal transmural perfusion gradient, which suggest subendocardial ischemia, have been associated with contractile dysfunction, transient LV cavity dilation, and an abnormal LVEF response. These findings reinforce the role of PET-based perfusion assessments as independent predictors of outcomes in HCM [93,94,95,96].

### 4.2. CMR Studies on Myocardial Perfusion in HCM

CMR allows for detailed myocardial tissue characterization, providing unique insights into CMD, myocardial hypertrophy, and fibrosis in HCM patients. Cardiac diffusion tensor imaging has confirmed the presence of cardiomyocyte disarray, a hallmark feature of HCM. Increased mean diffusivity, particularly in the subendocardium, has been observed, suggesting regional myocardial remodeling that may help explain subendocardial perfusion impairment [66].

Several CMR perfusion studies have demonstrated the complex relationship among CMD, ventricular hypertrophy, and myocardial fibrosis in HCM. HCM typically presents as a diffuse reduction in MBF, which is more pronounced in hypertrophic segments and in the subendocardium (Table 2). CMD is prevalent in a large proportion of HCM patients, with qualitative assessments indicating CMD in approximately 40% of cases and quantitative pixel-wise perfusion mapping revealing CMD in up to 80% of patients. CMD is more frequently associated with LV hypertrophy, non-sustained ventricular tachycardia, apical aneurysms, and the presence of LGE [62,63]. Although perfusion defects are more commonly observed in hypertrophied myocardial segments, they can also occur in regions with normal wall thickness, indicating widespread CMD in HCM [67]. These areas often exhibit higher T1 and T2 mapping values, increased ECV, and LGE, suggesting underlying tissue abnormalities and replacement fibrosis. In addition, impaired myocardial deformation parameters indicate compromised myocardial mechanics in these regions [67]. Interestingly, CMD has been observed even in morphologically normal myocardium, suggesting that it may serve as an early marker of disease progression before overt hypertrophy develops. However, more commonly, CMD leads to ischemic myocardial injury, contributing to both fibrosis and adverse cardiovascular outcomes, thereby highlighting its role as an independent risk factor in HCM [56,57]. A more pronounced pattern of regional myocardial microvascular dysfunction has been identified in HCM patients with LV outflow tract obstruction compared to those without obstruction, indicating that hemodynamic disturbances may play a role in worsening myocardial ischemia [58].

The use of myocardial transit time (MyoTT), a novel CMR parameter that reflects the passage of gadolinium contrast through the myocardial microvasculature, has demonstrated that the severity of CMD (measured using MyoTT) relative to extracellular matrix expansion (measured by native T1 and/or ECV mapping) is more pronounced in HCM than in cardiac amyloidosis. This finding suggests distinct pathophysiological mechanisms of microvascular impairment between the two conditions [97]. The assessment of genotype-phenotype differences in HCM using novel oxygen-sensitive CMR approaches has provided further insights. Studies utilizing perfusion and LGE techniques have confirmed that sarcomere mutation-positive HCM and variants of uncertain significance (VUS) exhibit blunted vasodilator stress myocardial perfusion and oxygenation responses compared to mutation-negative patients. Despite similar LGE burden across these groups, mutation-positive and VUS patients demonstrated greater CMD, reinforcing the genetic influence on microvascular pathophysiology in HCM [64]. In contrast, sarcomere mutation carriers without LV hypertrophy do not show significant impairments in stress myocardial perfusion, suggesting that CMD develops in later disease stages. However, oxygenation levels are already blunted in these individuals, indicating early metabolic abnormalities that precede overt hypertrophy [64,65].

Extensive myocardial replacement fibrosis, quantified using contrast-enhanced CMR, has been shown to improve risk stratification for SCD in HCM patients, particularly in individuals otherwise considered low-risk [98]. A strong association between LGE and CMD has been identified in most HCM patients [99]. MPR quantified by stress perfusion CMR is more impaired in HCM patients with LGE than in those without, indicating a strong link between fibrosis and ischemic burden [61]. However, a subset of patients with LGE does not exhibit perfusion abnormalities, suggesting that microvascular dysfunction and ischemia are not the sole contributors to fibrosis formation in HCM [99]. Adding complexity to the clinical picture, myocardial fibrosis detected via LGE can be responsible for rest perfusion defects in up to 30% of HCM patients, underscoring the need for high-resolution CMR techniques to distinguish ischemia from fibrotic changes [59]. Finally, myocardial replacement fibrosis is a key confounder when assessing ischemic burden in HCM. Areas of LGE are associated with significant reductions in MPR, which may lead to an overestimation of ischemic burden in uncorrected perfusion maps [60]. The use of high-resolution quantitative perfusion analysis with LGE correction enables the independent evaluation of microvascular ischemia and fibrosis, thereby improving risk stratification and prognostic assessment in HCM patients [60].

## 5. Cardiac Amyloidosis

Cardiac amyloidosis (CA) is a restrictive cardiomyopathy characterized by pseudo-hypertrophy resulting from the extracellular deposition of abnormal proteins in the myocardium [100]. While in past decades a positive biopsy was the only way to diagnose CA, the combination of several imaging modalities has recently been demonstrated to provide a non-invasive diagnosis of CA, thus restricting the indication for biopsy to those patients with equivocal or discordant findings [101]. According to the European Society of Cardiology (ESC) position paper, CMR can be used to implement the diagnostic algorithm of CA in both the “scintigraphy-based” and “laboratory-based” pathways, being particularly useful in patients with a positive hematologic test and negative scintigraphy (grade zero) [102]. Other recently published consensus documents have even proposed a CMR-based pathway for the diagnosis of CA [103].

CA is associated with significant alterations in myocardial perfusion, primarily due to extracellular amyloid deposition, vascular dysfunction, and microvascular impairment [71]. Histological studies have demonstrated that amyloid deposition affects not only the myocardial interstitium but also the intramyocardial arteries, leading to severe luminal narrowing, vascular disruption, and capillary rarefaction. These vascular abnormalities result in markedly reduced myocardial perfusion, even in the absence of epicardial coronary artery disease [70,71].

### 5.1. Non-CMR Studies on Myocardial Perfusion in CA

Quantitative MCE studies consistently demonstrate a reduction in total MBF and MBF reserve in patients with CA, highlighting the presence of CMD [104,105]. Doppler-based echocardiographic studies in mixed cohorts of wild-type transthyretin (ATTR) and light-chain (AL) amyloidosis patients further confirmed a significant reduction in coronary flow velocity reserve and longitudinal myocardial deformation reserve compared with healthy controls. This reduction correlated strongly with the physical capacity of CA patients, suggesting functional limitations secondary to myocardial ischemia and CMD [106].

SPECT-MPI in transthyretin amyloid cardiomyopathy has revealed distinct perfusion abnormalities that differ from those observed in CAD. While MPI remains a valuable tool for CAD assessment, its use in amyloidosis requires careful interpretation due to the pathophysiological changes induced by amyloid deposits, which may lead to hypoperfusion and misinterpretation of perfusion defects [107]. Dual SPECT imaging in ATTR patients has demonstrated a global reduction in myocardial perfusion, with regional heterogeneity predominantly affecting the septal wall, despite diffuse LV hypertrophy [108]. These findings suggest that perfusion abnormalities in amyloidosis are not solely due to increased wall thickness but rather reflect underlying microvascular dysfunction and amyloid-induced vascular remodeling.

Computed tomography perfusion (CTP) has emerged as a promising technique for evaluating myocardial perfusion and ECV in CA. CA patients exhibit higher ECV values, along with lower mean myocardial blood volume and MBF, compared to both non-amyloid cardiac hypertrophy and normal controls. These findings indicate that ECV and perfusion parameters obtained via CTP may serve as important prognostic markers, predicting mortality and adverse cardiovascular outcomes in CA patients [109].

N-13 ammonia PET studies comparing CA patients without epicardial CAD to patients with hypertensive LV hypertrophy have demonstrated lower resting and stress MBF, along with higher minimal coronary vascular resistance in the amyloidosis cohort. Remarkably, more than 95% of CA patients exhibited a severely reduced peak stress MBF (<1.3 mL/g/min), confirming that CMD is highly prevalent in CA and likely explains the presence of anginal symptoms even in the absence of significant epicardial CAD [110]. The combination of dual-tracer PET imaging (11C-acetate and 15O-water) with echocardiographic or invasive RV pressure measurements has demonstrated that RV MBF reserve correlates directly with RV systolic pressures, while LV MBF is inversely correlated with wall thickness. Moreover, an imbalance between RV and LV myocardial perfusion (RV-MBF/LV-MBF > 56%) has been associated with higher N-terminal pro-B-type natriuretic peptide levels, worsening New York Heart Association functional class, increased RV afterload (pulmonary hypertension), and a higher risk of adverse cardiovascular events [111].

### 5.2. CMR Studies on Myocardial Perfusion in CA

CMR-based quantitative stress MBF mapping has confirmed the presence of severe inducible myocardial ischemia in CA, with MPR values comparable to those seen in patients with three-vessel CAD [70,71]. CA typically presents as a severe diffuse perfusion impairment, characterized by an endocardial–epicardial transmural gradient and a base-to-apex longitudinal gradient that correspond to amyloid infiltration (Table 2). In patients with AL amyloidosis, first-pass perfusion studies have identified distinct regional microvascular dysfunction, including lower first-pass perfusion upslope, prolonged time to maximum signal intensity, and lower maximum signal intensity. These perfusion abnormalities were more pronounced in patients with impaired systolic function compared to those with preserved LVEF [68].

MPR has also been linked to fibrosis burden, as demonstrated by LGE quantification. Patients with transmural LGE exhibit significantly lower MPR than those with subendocardial or no LGE, reinforcing the association between microvascular dysfunction, fibrosis progression, and worse outcomes. Interestingly, survivors with CA tend to have lower ECV but higher stress MBF and MPR, indicating that patients with a higher amyloid burden (elevated ECV) and lower MPR have a poorer prognosis [73].

Rest myocardial perfusion in CA has been shown to correlate directly with blood biomarkers, particularly troponin levels, while being inversely correlated with amyloid burden, as measured by ECV fraction. However, no significant correlation has been observed between native T1 mapping values and resting perfusion, suggesting that T1 mapping alone may not fully capture perfusion abnormalities in CA [112]. In contrast, stress MBF and MPR have been identified as early disease markers, particularly in patients with subclinical amyloid infiltration (elevated ECV but no LGE). These perfusion markers have been found to correlate with native T1 mapping but not with T2 mapping, further supporting the role of MPR as a functional parameter of early amyloid-induced vascular dysfunction [69].

The integration of multiparametric CMR techniques has significantly improved our understanding of the mechanisms driving systolic dysfunction in CA, which is widely regarded as a key prognostic marker in ATTR-CM. Quantitative stress perfusion CMR has been increasingly utilized to evaluate CMD in CA. These assessments provide incremental prognostic value, particularly in characterizing systolic dysfunction and differentiating risk profiles in the advanced stages of the disease [73]. One of the major contributors to cardiac dysfunction in CA is ECV expansion, a surrogate marker of amyloid burden and disease progression. ECV expansion contributes to reduced myocardial work efficiency, which is estimated by external cardiac work (a product of stroke volume and mean arterial pressure normalized to LV myocardial mass). These structural and vascular changes ultimately lead to progressive cardiac dysfunction [72]. A strong association between ECV and myocardial work efficiency has been reported, with adverse interstitial remodeling (assessed using ECV) exerting a direct impact on myocardial performance. Additionally, a smaller indirect effect of resting MBF on myocardial work efficiency has been observed, further supporting the concept that amyloid-induced interstitial expansion is the dominant factor contributing to functional deterioration [72].

Combining CMR and nuclear modalities enhances both diagnostic accuracy and prognostication. CMR excels at mapping amyloid burden via characteristic diffuse subendocardial LGE and elevated ECV, while fully quantitative perfusion mapping still lacks complete standardization and might be affected by contrast saturation artifacts. PET-derived MBF and CFR measurements not only validate CMR perfusion abnormalities but also help distinguish vascular from interstitial contributions to dysfunction. In settings without PET, dual-energy CT perfusion might offer simultaneous ECV and MBF estimation, while contrast echocardiography provides bedside assessment of microvascular reserve. A streamlined multimodality approach—leveraging CMR tissue characterization, PET quantitative flow, SPECT/CT broad availability, and echocardiography portability—yields the most comprehensive evaluation of micro-vascular dysfunction in CA, informing risk stratification and therapy monitoring.

## 6. Arrhythmogenic Cardiomyopathy

Arrhythmogenic cardiomyopathy (ACM) is a genetically determined heart muscle disease characterized by fibro-fatty myocardial replacement and is clinically associated with malignant ventricular arrhythmias and SCD. Although it was originally described as a RV-predominant disorder, the growing recognition of biventricular and left-dominant phenotypic variants has broadened the concept of ACM to include both RV and LV involvement [113].

CMR has long been considered a non-invasive gold standard imaging modality for identifying morpho-functional abnormalities in ACM. With the publication of the updated European Task Force Criteria [114], CMR has gained even greater diagnostic relevance, as LGE patterns are now included among the structural diagnostic criteria for ACM. LGE enables the detection of myocardial fibrosis and fatty infiltration, providing crucial insights into disease severity, risk stratification, and prognosis [79]. Given its high spatial resolution, CMR can detect subtle myocardial abnormalities that may be present even before overt structural dysfunction or arrhythmias develop.

Beyond the classical genetic and structural framework of ACM, recent evidence has identified a subgroup of patients presenting with active myocardial inflammation, termed arrhythmogenic inflammatory cardiomyopathy (AIC) [115]. The identification of these patients is key, since the aetiology of their arrhythmic burden is likely related to both scar-mediated and direct inflammatory mechanisms, which may require different treatment approaches [115]. The concept of “hot phases” in ACM has also emerged to describe episodes of acute myocardial inflammation that may occur independently of genetic predisposition or fibro-fatty replacement [116]. These phases are characterized by edema, immune cell infiltration, and increased metabolic activity, which contribute to arrhythmogenesis through mechanisms distinct from fibrosis-related reentrant circuits [116].

Recent evidence highlights the role of myocardial perfusion abnormalities in ACM, further supporting its complex pathophysiology. Perfusion abnormalities are usually heterogeneous, often corresponding to fibrotic and inflamed areas (Table 2). In a study by Tung et al., PET and perfusion CMR revealed that nearly half of patients with nonischemic cardiomyopathy and ventricular arrhythmias exhibited myocardial inflammation and perfusion abnormalities [74]. These findings suggest that in addition to fibrotic remodeling, microvascular dysfunction and inflammatory processes contribute to arrhythmogenesis in ACM [74]. Additionally, areas of perfusion–metabolism mismatch, characterized by inflammation either in the absence (“early ACM”) or in the presence of fibrosis (“late ACM”), are associated with a higher risk of recurrent ventricular tachycardia and adverse outcomes [74]. This aligns with evidence indicating that patients with concurrent perfusion defects and inflammatory activity experience a more aggressive disease course, characterized by an increased arrhythmic burden despite medical or ablation therapy.

In the future, the integration of LGE, perfusion imaging, and myocardial inflammation assessment through PET/CMR hybrid imaging could enhance risk stratification and guide therapeutic interventions in ACM patients.

## 7. Fabry Disease

Fabry disease (FD) is an X-linked lysosomal storage disorder caused by a deficiency of the enzyme alpha-galactosidase A. This deficiency leads to the accumulation of glycosphingolipids within lysosomes in various tissues, including the myocardium, resulting in a hypertrophic cardiomyopathy phenotype [117]. Multiparametric CMR has emerged as a crucial tool in the assessment of FD, as it allows for the detection of different stages of myocardial involvement [117]. The earliest detectable abnormality is sphingolipid storage, which manifests as low myocardial T1 values on native T1 mapping. As the disease progresses, the development of LV hypertrophy is accompanied by focal inflammation and fibrosis, which typically begin in the basal inferolateral wall and can be detected using LGE. T2 mapping further enables the identification of edema, which correlates with active inflammation and myocyte injury. Beyond structural remodeling, endothelial storage in FD has been implicated in microvascular dysfunction, but its role in the development of myocardial perfusion abnormalities has not been fully characterized [117].

Coronary microvascular function is markedly impaired in Fabry patients, irrespective of LV hypertrophy and gender. In a study involving 30 Fabry patients and 24 healthy controls, MBF during dipyridamole infusion with 13N-labelled ammonia by PET was reduced in all patients compared with controls (1.8 ± 0.5 and 3.2 ± 0.5 mL/min/g, respectively, *p* < 0.001). Moreover, flow impairment was most severe in patients with LV hypertrophy (1.4 ± 0.5 mL/min/g in males and 1.9 ± 0.5 mL/min/g in females) but was also evident in those without LV hypertrophy (1.8 ± 0.3 mL/min/g in males and 2.1 ± 0.4 mL/min/g in females) [118].

A recent study using quantitative CMR perfusion mapping investigated MBF in FD patients [75] (Table 2). Compared with controls, FD patients exhibited significantly reduced global stress MBF, even in the absence of LV hypertrophy, suggesting that CMD occurs early in the disease process. The impairment in perfusion was more pronounced in patients with LV hypertrophy, indicating that microvascular dysfunction may progress in parallel with structural remodeling [119]. Further analysis revealed a transmural perfusion gradient, with greater perfusion impairment in the subendocardial layer compared to the epicardium, particularly in patients with LV hypertrophy. This pattern is consistent with findings in other hypertrophic cardiomyopathies, where subendocardial ischemia is a hallmark of microvascular dysfunction. The presence of LGE and lower myocardial T1 values, indicative of fibrosis and sphingolipid accumulation, respectively, were independently associated with reduced stress MBF [119]. This suggests that microvascular dysfunction is linked to both storage and the progression of fibrosis, reinforcing the hypothesis that ischemia may contribute to the transition from inflammation to irreversible myocardial damage.

## 8. Limitations of CMR

CMR requires a dedicated cardiac scanner and specialized expertise, making it less widely available compared to echocardiography. The higher costs associated with CMR, along with the need for precise operator skills, limit its accessibility, particularly in resource-constrained settings.

Technical limitations may also arise in specific patient populations. Tachyarrhythmias and breathing artifacts can affect image quality, although these issues are often manageable with advanced imaging protocols. Claustrophobia remains a relative contraindication, although selected patients may tolerate the procedure with mild sedation or general anesthesia if necessary. The presence of metallic implants, including pacemakers and defibrillators, has traditionally posed a major limitation for CMR. However, the development of CMR-conditional devices has enabled safe imaging in selected patients, provided that appropriate device reprogramming is performed before the scan [120,121].

The use of gadolinium-based contrast agents in patients with severely impaired renal function remains a significant limitation. Although the risk of nephrogenic systemic fibrosis is extremely low with newer cyclic gadolinium-based contrast agents, these contrast agents are generally contraindicated in individuals with a glomerular filtration rate below 30 mL/min/1.73 m^2^ [122]. In such cases, non-contrast imaging techniques, including T1 and T2 mapping, as well as oxygenation-sensitive techniques, may provide valuable diagnostic information without the need for contrast administration [123,124].

In contrast to the high accuracy and reliability of CMR in evaluating cardiac function and volumes, perfusion CMR is adversely affected by multiple potential factors during data acquisition and post-processing. Various image acquisition techniques, various contrast agents and doses, variable blood flow responses to stress agents, and diverse post-processing algorithms all represent significant sources of variability.

The intra- and inter-observer reproducibility of a semi-quantitative estimation of MPR using commercially available software from time–intensity curves acquired at rest and during adenosine stress has been demonstrated to be suboptimal, with intraclass correlation coefficients of 0.89 and 0.80, respectively [125]. Moreover, quantitative myocardial perfusion with CMR is even more technically challenging and depends on several correction algorithms, which can lead to significant variability in the MBF values. The correlation between simultaneous MBF measurements using dynamic contrast-enhanced CMR with simultaneous ^15^O-water PET as a reference, both at rest and during adenosine infusion in a fully integrated PET-MR scanner, was good, with negligible bias between CMR-based and PET-based MBF values. However, there was considerable variability, especially for the higher MBF values [126]. On the other hand, a recent study involving 42 healthy volunteers who underwent fully automated, inline myocardial perfusion mapping through two separate CMR scans showed good repeatability, especially for both rest and stress MBF, while repeatability was still suboptimal for MPR, particularly in regional analysis [127].

Developing a standardized, reliable, and accurate tool for CMR perfusion analysis remains a challenge; achieving this would greatly strengthen the role of perfusion CMR in enhancing clinical decision-making and patient care. Despite these limitations, ongoing advancements in hardware, new design sequences, imaging protocols, and contrast agent safety continue to improve the feasibility and diagnostic accuracy of CMR, expanding its clinical utility across a broader range of patients.

## 9. Future Directions for Myocardial Perfusion in CMR

Several novel experimental CMR sequences are currently under investigation, primarily in the research phase, though they have not yet been widely adopted in routine clinical practice. These advanced techniques aim to improve spatial and temporal resolution, motion robustness, and quantification accuracy, potentially enhancing the clinical utility of perfusion CMR in the coming years. One promising area of development involves advanced accelerated k-t imaging techniques, such as k-t SENSitivity Encoding (SENSE) and k-t principal component analysis (PCA), which utilize spatial–temporal correlations to expedite image acquisition while preserving resolution. These methods improve the temporal fidelity of dynamic magnetic resonance by decomposing both training and undersampled data into optimized temporal basis functions, leading to enhanced reconstruction accuracy [128].

Although not yet fully integrated into standard clinical workflows, compressed sensing perfusion CMR techniques offer another approach for highly undersampled data acquisition while preserving image quality. By exploiting data sparsity, compressed sensing enables high-resolution perfusion imaging within a single heartbeat. When combined with parallel imaging, it may provide up to an eight-fold acceleration in vivo, achieving whole-heart coverage with high spatial and temporal resolution using standard coil arrays [129].

The development of dual-sequence and simultaneous multi-slice perfusion (SMS) imaging represents another avenue aimed at improving myocardial coverage and imaging efficiency. These approaches allow for the acquisition of multiple slices simultaneously without sacrificing in-plane spatial resolution. To maximize the signal-to-noise ratio, SMS can be combined with a balanced steady-state free precession readout. The use of gradient-controlled local Larmor adjustment helps counteract off-resonance artifacts, while iterative reconstruction with spatial and temporal regularization mitigates signal-to-noise ratio loss [8].

Advancements in motion correction and free-breathing perfusion imaging have led to the development of self-gated perfusion sequences that employ self-navigation and respiratory motion correction. These approaches eliminate the need for breath-holding, making them particularly useful for patients with respiratory limitations. In normal subjects, self-gated perfusion sequences have demonstrated excellent agreement with conventional ECG-gated methods, particularly during systolic phases, where perfusion measurements appear more consistent than in diastole [130].

The introduction of low-rank motion compensation (LRMC) reconstructions has also significantly improved image quality for accelerated free-breathing first-pass MPI. LRMC techniques estimate beat-to-beat nonrigid respiratory motion directly from acquired data and incorporate it into low-rank reconstructions, outperforming previous iterative SENSE and low-rank plus sparse methods [131].

The evolution of 3D and 4D whole-heart perfusion imaging represents another major advancement in overcoming the spatial coverage limitations of standard 2D techniques. By capturing volumetric data across multiple time points, 3D and 4D imaging eliminate the need for geometric assumptions in ischemic tissue quantification [132] and provide full left ventricular coverage in a single acquisition, thereby reducing slice misregistration. A combination of compressed sensing, parallel imaging, and golden-angle radial sampling has enabled the development of iterative golden-angle radial sparse parallel magnetic resonance imaging (iGRASP), which enhances motion robustness and improves spatial–temporal resolution while minimizing artifacts [133].

Among non-contrast approaches, oxygenation-sensitive CMR allows for the noninvasive in vivo assessment of myocardial tissue oxygenation by exploiting the paramagnetic properties of deoxyhemoglobin. As hemoglobin saturation increases, the blood oxygenation level-dependent signal is enhanced, which can be detected using specific T2 or T2* sequences [124]. Other experimental non-contrast CMR techniques for the assessment of myocardial perfusion include native T1 mapping, which is sensitive to myocardial hyperemia and increases during vasodilation, and arterial spin labeling, which labels the water protons in arterial blood with a magnetic tag, allowing for the measurement of blood flow by tracking these “tagged” protons as they circulate.

Further innovations in hyperpolarized contrast agents have led to the development of MRS and imaging approaches as alternatives to gadolinium-based contrast agents. Carbon-13 (^13^C) and Fluorine-19 (^19^F) CMR techniques are among the most promising of these novel imaging strategies. Unlike conventional hydrogen-based imaging, these approaches rely on specific isotope properties, requiring dedicated hardware, coils, and rapid pulse sequences. The use of hyperpolarized pyruvate tracers has enabled real-time in vivo assessment of myocardial metabolism under different physiological and pathological conditions [134,135,136].

In 3D whole-heart coronary CMR techniques, tissue contrast and arterial visualization have been improved. Unlike 2D techniques, 3D imaging provides volumetric acquisition of truly contiguous thin partitions, capturing the entire coronary arterial tree in a single scan while minimizing reliance on slice orientation. Experimental sequences and specialized coils have been tested in animal models to enhance high-resolution intravascular coronary imaging [137].

The development of 4D flow CMR has expanded the ability to quantify MBF and coronary resistance. This is achieved through reformatted phase–contrast planes, which allow for detailed flow measurements via the coronary sinus, offering new insights into coronary hemodynamics and microvascular function [138].

In recent years, artificial intelligence (AI) and machine learning have been introduced into CMR-based perfusion quantification, offering automated MBF assessments with enhanced accuracy and reduced interobserver variability. A major limitation of advanced image reconstruction algorithms is that they iteratively solve a nonlinear optimization problem, resulting in lengthy computation times. Additionally, these algorithms treat each new image dataset as an entirely separate reconstruction task, without leveraging information from previous reconstructions. AI offers a potential way to overcome some of these constraints by using a trained model that can learn to map raw data directly to images. This approach allows the model to incorporate knowledge gained from a diverse set of patients during training. Once trained, the reconstruction process becomes significantly faster, requiring only a single forward pass through the neural network to generate new images [139].

AI initially focused on automating image processing tasks, such as anatomical segmentation and landmark detection, which are crucial steps toward streamlining workflows. This makes the method user-independent and speeds up data analysis [139]. AI-driven models can also assist in automated MBF quantification, although these techniques remain in the developmental phase and are not yet standard in clinical practice [119]. Additionally, deep learning-based reconstruction and motion correction algorithms have enabled high-resolution image generation from undersampled datasets, reducing overall scan times while maintaining diagnostic accuracy [140]. Future developments using AI are expected to focus on optimizing image acquisition, improving image quality and quantification, shortening scan times, and enhancing interpretation through automated reporting. The next wave of research will need to prioritize the development of AI systems that are robust, reproducible, and generalizable, with a stronger emphasis on clinical validation, including trials in prospective clinical settings.

While some of these novel perfusion techniques, such as perfusion mapping, are beginning to see clinical implementation, many advanced CMR methodologies remain experimental and are not yet part of routine clinical practice. The future integration of multiparametric imaging, AI-driven quantification, machine learning for motion correction and image reconstruction, and novel contrast agents holds great potential for further enhancing myocardial perfusion assessment and broadening the clinical applications of CMR in both ischemic and non-ischemic heart disease.

## 10. Conclusions

CMR has revolutionized the assessment of nonischemic cardiomyopathies by providing detailed myocardial tissue characterization and uncovering microvascular dysfunction, a key contributor to disease progression. Beyond ruling out ischemia, CMR perfusion imaging offers valuable prognostic insights that aid in risk stratification and personalized management. In the future, myocardial perfusion imaging might help detect early subclinical impairment of both macro- and microvascular function, as well as track the effects of pharmacological and interventional therapies.

Recent advancements, including high-resolution quantitative perfusion imaging, AI-driven analysis, and motion-corrected techniques, have enhanced diagnostic accuracy and reproducibility. However, widespread clinical adoption requires further validation and standardization. As technology continues to evolve, emerging techniques such as deep learning-based image reconstruction and 4D whole-heart perfusion imaging hold promise for refining myocardial perfusion assessment. Integrating these innovations into clinical workflows will further establish CMR as an indispensable tool for diagnosing and managing nonischemic cardiomyopathies, ultimately improving patient outcomes.

## Figures and Tables

**Table 1:** Comparison of different cardiovascular imaging modalities for the non-invasive assessment of cardiac anatomy, function, tissue characterization, and myocardial perfusion.

	TTE	CMR	SPECT/PET	CT/PC-CT
**Technical characteristics**				
Availability	**+++**	**+**	**++/−**	**++/−**
Spatial resolution (mm)	0.5–1	1–2	4–8/5–15	0.5/0.1–0.2
Temporal resolution (ms)	<10	20–50	100–300	80–135/50–100
Radiation exposure	−	−	+++/++	+++/++
Cost	€	€€€	€€/€€€	€€/€€€
**Cardiac morphology and function**				
Chamber volume	**++**	**+++**	**+**	**++/+++**
Wall thickness	**++**	**+++**	**−**	**++/+++**
Systolic function	**++**	**+++**	**+**	**+/++**
Diastolic function	**+++**	**++**	**+**	**+/++**
**Myocardial tissue characterization**				
Inflammation	**−**	**+++**	**+++**	**−**
Fibrosis	**+**	**+++**	**+**	**+/++**
Amyloidosis	**+**	**+++**	**+++**	**+/++**
Metabolism	**−**	++ (MRS)	**+++** (FDG-PET)	**−**
Oxygenation	**−**	**++** (OS-CMR)	**−**	**−**
**Myocardial perfusion and coronary artery disease**				
Coronary anatomy	+/− (Coronary origin)	+ (Coronary origin)	−	+++
Coronary flow (noncontrast)	++ (Doppler in LAD)	+ (CS phase contrast)	−	−
Contrast for perfusion	Microbubbles	Gadolinium	^99m^Tc, ^201^Tl/ ^82^Rb, ^13^N-NH_3_, ^15^O-H_2_O, ^18^F-flurpiridaz	Iodine
Contrast safety limitations	Headache (rare) Paresthesia (rare) Nausea (rare) Allergy (very rare)	Nausea (rare) Allergy (very rare) Nefrogenic sclerosis (exceptional, only few patients with GFR < 30 mL/min exposed to old linear chelates)	Radioactivity	Nausea (rare) Allergy (rare) Thyreotoxicosis (rare) Renal failure (rare, only in patients with GFR <30 mL/min)
Qualitative perfusion	+	+++	++/+++	++
Semiquantitative perfusion	+	++	++/+++	+
Quantitative perfusion	−	+++	+/+++	++
Microvascular assessment	+	++	++	+
Cycloergometer stress	+++	− (experimental)	+++	− (experimental)
Pharmacological stress	Vasodilator (dipyridamole, adenosine, regadenoson), dobutamine in selected cases			
**Feasibility in patients with**				
Severe renal failure	+++	+	+++	−
Arrhythmias	+++	+	++	+/++
Haemodynamic instability	+++	−	−	++
Pacemaker/defibrillator	+++	+	+++	++/+++
Claustrophobia	+++	+	+++	++
COPD	+	+++	+++	+++
Obesity	+	++	++	++/+++
Pregnancy	+++	++	−	−

CMR, cardiac magnetic resonance; COPD, chronic obstructive pulmonary disease; CS, coronary sinus; CT, computed tomography; GFR, glomerular filtration rate; FDG, fluoro-deoxy-glucose; LAD, left anterior descending; MRS, magnetic resonance spectroscopy; OS, oxygenation sensitive; PC-CT, photon counting compute tomography; PET, positron emission tomography; SPECT, single-photon emission computed tomography; TTE, transthoracic echocardiography. The symbols “−, +, ++, +++” represent a semiquantitative score to indicate the pros and cons for each modality.

## Abbreviations

AIF	Arterial input function
ACM	Arrhythmogenic cardiomyopathy
AL	Light-chain amyloidosis
ATTR	Transthyretin amyloidosis
AVB	Atrioventricular block
CA	Cardiac amyloidosis
CAD	Coronary artery disease
CFR	Coronary flow reserve
CMD	Coronary microvascular dysfunction
CMR	Cardiovascular magnetic resonance
CTP	Computed tomography perfusion
DCM	Dilated cardiomyopathy
DPD	3,3-Diphosphono-1,2-propanodicarboxylic acid (bone-avid SPECT tracer)
ECV	Extracellular volume
FD	Fabry disease
FGE	Fast gradient-echo
HCM	Hypertrophic cardiomyopathy
HF	heart failure
ICD	Implantable cardioverter–defibrillator
iGRASP	Iterative golden-angle radial sparse parallel magnetic resonance imaging
k-t PCA	Principal component analysis
k-t SENSE	SENSitivity Endocing
LAD	Left anterior descending
LGE	Late gadolinium enhancement
LRMC	Low-rank motion compensation
LV	Left ventricle
LVEF	Left ventricular ejection fraction
MBF	Myocardial blood flow
MCE	Myocardial contrast echocardiography
MPI	Myocardial perfusion reserve
MPR	Myocardial perfusion reserve
MPRI	Myocardial perfusion reserve index
MRS	Magnetic resonance spectroscopy
MyoTT	Myocardial transit-time
PET	Positron emission tomography
PYP	Pyrophosphate (Tc-99m-PYP SPECT tracer)
RV	Right ventricle
SCD	Sudden cardiac death
SPECT	Single-photon emission computed tomography
SSFP	Steady-state free precession
Tc-99m	Technetium-99m
SMS	Simultaneous multi-slice imaging
VUS	Variance of uncertain significance
